# Implementation, Mechanisms and Context of the MAMAACT Intervention to Reduce Ethnic and Social Disparity in Stillbirth and Infant Health

**DOI:** 10.3390/ijerph18168583

**Published:** 2021-08-14

**Authors:** Trine Damsted Rasmussen, Helle Johnsen, Signe Smith Jervelund, Ulla Christensen, Anne-Marie Nybo Andersen, Sarah Fredsted Villadsen

**Affiliations:** 1 Section of Epidemiology, Department of Public Health, University of Copenhagen, Øster Farimagsgade 5, P.O. Box 2099, 1014 Copenhagen, Denmark; amny@sund.ku.dk; 2 Department of Midwifery and Therapeutic Sciences, University College Copenhagen, Sigurdsgade 26, 2200 Copenhagen, Denmark; HEJO@kp.dk; 3 Section for Health Services Research, Department of Public Health, University of Copenhagen, Øster Farimagsgade 5, P.O. Box 2099, 1014 Copenhagen, Denmark; ssj@sund.ku.dk; 4 Section of Social Medicine, Department of Public Health, University of Copenhagen, Øster Farimagsgade 5, P.O. Box 2099, 1014 Copenhagen, Denmark; ulch@sund.ku.dk (U.C.); sfv@sund.ku.dk (S.F.V.)

**Keywords:** complex interventions, process evaluation, antenatal care, pregnancy complications, ethnicity, immigrants, disadvantaged groups, disparities, cultural competence, health literacy

## Abstract

The MAMAACT intervention aimed to address ethnic and social disparity in stillbirth and infant health by improving management of pregnancy complications. This process evaluation of the intervention was guided by the British Medical Research Council’s framework. We examined implementation through dose, reach, and fidelity, important mechanisms and the influence of contextual factors. The intervention included a six-hour training session for antenatal care (ANC) midwives in intercultural communication and cultural competence, two follow-up dialogue meetings, and health education materials (leaflet and app) on warning signs of severe pregnancy complications and how to respond for pregnant women. A mixed-methods approach was applied. Cross-sectional survey data and administrative data were used to assess intervention reach and dose. Qualitative data (records from dialogue meetings with midwives, participant observations and field notes from ANC visits, focus group interviews with midwives, and individual interviews with non-Western immigrant women) evaluated intervention fidelity, mechanisms, and contextual barriers. More than 80% of women received the MAMAACT leaflet and many found the content useful. The app was used more selectively. Midwives described being more aware and reflective in their communication with women from various cultural backgrounds. Organizational factors in ANC (time pressure, lack of flexibility in visits, poor interpreter services), barriers in women’s everyday life (lack of social network, previous negative experiences/lack of trust and domestic responsibilities), and habitual interaction patterns among midwives served as contextual barriers. The reach of the intervention was high and it was evaluated positively by both pregnant women and midwives. Organizational factors hindered changes towards more needs-based communication in ANC potentially hindering the intended mechanisms of the intervention. When interpreting the intervention effects, attention should be drawn to both organizational and interpersonal factors in the clinic as well as the pregnant women’s life situations.

## 1. Introduction

Deaths in early life are tragic events. Ethnic and social disparities in stillbirth and infant death can be seen as the tip of the iceberg of the health inequities later in life [1]. Across Europe, immigrant women are found to have an increased risk of stillbirth and infant death compared to non-immigrant women [2,3], although findings also reflect heterogeneity [4]. Likewise, social disparity is documented in stillbirth [5,6]. The underlying causes of the increased risk are complex and still not fully understood [7] but a contributing cause is suboptimal maternity care [5]. For immigrant women miscommunication, language barriers, and delays in seeking and receiving care that induce increased severity of pregnancy complications [8,9,10] are also highlighted as causes of suboptimal care [4,11,12]. Thus, mechanisms for differential care have been linked to barriers in interpersonal interactions between users and providers in healthcare encounters [13,14]. To overcome the barriers for immigrant and socially disadvantaged women in receiving optimal maternity care, also in case of pregnancy complications, it is important to improve the interaction and communication between the maternity care providers and the women. To mitigate the health disparities from a structural level, it has been suggested to increase reflection among healthcare providers on how social and cultural differences in the encounters affect the trust and interaction [14,15,16] as well as increase responsiveness to the diversity of the users’ health literacy levels [17,18]. Nevertheless, a recent systematic review found no interventions to improve health literacy for pregnant women [19] showing a need for more research and action in this field.

In Denmark, the MAMAACT intervention was developed to reduce ethnic and social disparity in stillbirth and infant health [6,20] by improved management of pregnancy complications [21]. Initially, a mixed-method needs assessment was conducted and showed gaps in the midwifery-based antenatal care’s (ANC) active engagement of women, attention to the individual needs and diversity of women, communication about signs of pregnancy complications, and how to navigate the maternity care pathways [21]. A feasibility study showed that midwives found the intervention relevant and acceptable [22], and after undergoing a few adaptations, it was implemented in a national cluster randomized trial. All 20 maternity wards in Denmark were invited to participate and 19 joined. Using simple randomization, ten wards (each ward representing a cluster) were randomized to the intervention group and nine to the control group.

The intervention was developed as a universal intervention aiming to address the issues of both ethnic and social disparities in early life mortality. However, the intervention components and the data sampling had their focal point on challenges faced by ethnic minority women. These aspects were assessed to also have an impact on socially disadvantaged women measured by low education level with primary education or lower as the highest attained education. Ethnicity was measured using country of birth and immigrant women were defined by being born abroad to foreign-born parents in accordance with Statistics Denmark’s definition [23]. In this study, we focused on the group of women born in non-Western countries.

The hypothesis of MAMAACT was that training midwives in cultural competence and intercultural communication and introducing health education materials about when to react to body symptoms and where to go in the healthcare system among pregnant women, would improve communication between pregnant women and midwives regarding pregnancy complications, and thus improve care and perinatal health in the disadvantaged groups. So, the MAMAACT intervention is a structural intervention at the ANC level.

**Figure 1:**
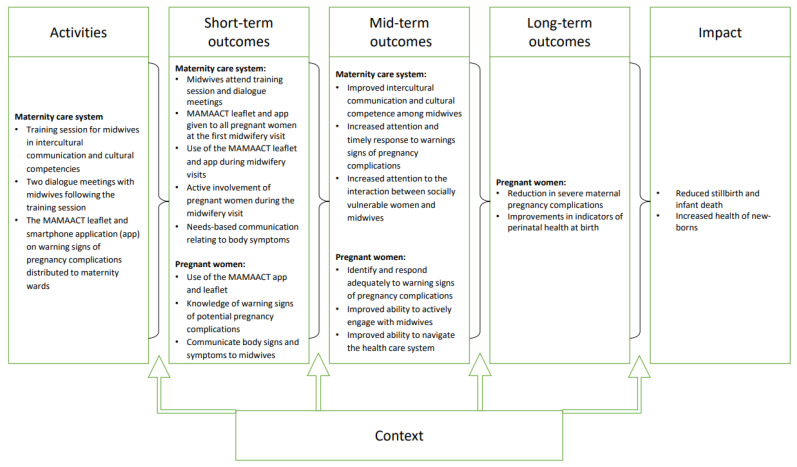
The MAMAACT logic model illustrating the intervention activities and expected outcomes.

The intervention development was further based on theories of cultural competence [15,16] and cultural health capital [14], and certain domains of health literacy [24] to guide interpersonal interaction between midwives and pregnant women and health system navigation. Following the British Medical Research Council’s (MRC) guidance [25,26], a logic model was developed to illustrate the intervention activities, the expected outcomes, and future impact (Figure 1). As stillbirth and infant deaths are rare events in Denmark, the trial would not have statistical power to show effects on these outcomes. Guided by the hypothesized relationship between intervention activities and study outcomes as depicted in the logic model the selected primary trial outcome was to increase active engagement with healthcare providers (a specific domain of health literacy [24], measured among pregnant women, but interpreted as an indicator of the health literacy responsiveness of the midwives [17]). Secondary outcomes were to improve health system navigation, knowledge about pregnancy complications among women, a reduction in severe maternal pregnancy complications and improvements in indicators of perinatal health at birth. The trial followed the principles of proportional universalism [27] and was implemented for all women, but emphasizing the need for midwives to center care around the women with the greatest need for special attention.

In complex intervention research, it has been acknowledged that interventions that aim to improve health, often work through changed social practices, which are sensitive to context [28,29]. Thus, the effect of a complex intervention will be a result of how the intervention was received and adapted to the specific context. Local adaption is considered needed to activate the mechanisms of change for the specific agents delivering or receiving the intervention. Accordingly, MRC underlines the importance of process evaluations within trials and provides guidance on how to analyze the process of implementation and the role of context [25]. Thus, important contextual factors that hinder or facilitate implementation, as well as unintended effects, can be identified and combining quantitative and qualitative methods in this process is warranted. The current study is a mixed-method process evaluation of the MAMAACT intervention. Using in-depth qualitative data from five of the ten intervention units, we have previously analyzed specific elements of the intervention [30,31,32]. However, these analyses zoomed in on specific mechanisms and contextual factors and did not provide a comprehensive assessment of whether the intervention as such was implemented as intended. Therefore, drawing on multiple data sources including extracts from these studies by Johnsen et al., we now gain a comprehensive process evaluation according to the MRC framework, assessing all elements of the activities, and short and midterm outcomes as given in the logic model. This enable reflections on how the MAMAACT health literacy responsiveness intervention is relevant to new contexts in efforts to reduce social and ethnic disparities in early life.

### Aims

The aims of the process evaluation were:

- To analyze the implementation of the MAMAACT intervention in relation to which elements were implemented and how (dose, reach, and fidelity);
- To identify important mechanisms of impact by examining participants’ responses to and interactions with the intervention;
- To analyze how contextual factors enabled or hindered the implementation process and the intended mechanisms and outcomes of the intervention.

## 2. The Framework of the Intervention

### 2.1. Setting

In Denmark, ANC is publicly funded and free of charge, and women are entitled to receive at least five free ANC visits [33]. The provision of health education on pregnancy complications is a central aspect of the national Danish policy [34] for ANC. Danish ANC is differentiated according to the women’s needs and divided into four levels ranging from basic ANC provision to extended ANC provision involving interdisciplinary cooperation [34]. Basic ANC is shared between general practitioners (GPs) and midwives. The purpose of the visits to the GP is to assess the physical and psychosocial health of the pregnant woman and to consider if specialized care is required. In uncomplicated pregnancies, the midwife is the maternity care provider the woman sees the most.

### 2.2. The MAMAACT Intervention and the Intended Implementation

The intervention included the following components:

- A six-hour training session for all midwives working in ANC in intercultural communication and cultural competence;
- Two one-hour follow-up dialogue meetings with ANC midwives;
- Health education materials (leaflet and smartphone app) on warning signs of severe pregnancy complications and how to respond for pregnant women.

#### 2.2.1. Training Session in Intercultural Communication and Competence

The training session was facilitated by the MAMAACT project team (including three of the authors: TDR, AMNA, SFV) in cooperation with specialists from two Danish Migrant Health Clinics. The training was structured around Seeleman et al.’s concept of cultural competence [16] and included scientific knowledge of the increased risk of poor reproductive outcomes among some immigrant groups in Denmark [21]. A communication module taught principles of ANC communication and intercultural communication including casework to strengthen skills in practice. Specialists from the Migrant Health Clinics facilitated a case-based discussion on intercultural communication. They used everyday experiences from the clinics to help midwives reflect on their health-related implicit cultural assumptions and experiences. The last part of the training session introduced the midwives to the leaflet and app and how to integrate these in ANC. The training session was held ten times at different locations across Denmark in October–November 2018. All maternity wards appointed a project midwife, who received a full-day introductory course and acted as the communication link between the research project and the hospital. The project midwife was compensated for the project work and the ward was compensated a fixed amount per hour equivalent to the day-hour salary of midwives for every midwife attending the post graduate training (training session and dialogue meetings).

#### 2.2.2. The Dialogue Meetings

The dialogue meetings served as a tool for sharing experiences with colleagues and reflections on how to integrate and further develop insights from the training session into daily practice. The meetings were facilitated by the local project midwife and guided by a written manual and occurred in two rounds so that the full training for each midwife was the training session and two dialogue meetings. At the first round of the dialogue meetings, midwives were to bring a case based on their own experience of using the leaflet in the communication with pregnant women, however, this proved challenging. For the second round, the manual was revised and a specific case reflecting challenges faced by immigrant women in the interaction with the midwifery-based ANC was developed together with the MAMAACT user board consisting of a non-profit organization (Neighbourhood Mothers) [35] to support reflection on intercultural communication.

#### 2.2.3. The Leaflet and App

Descriptions in both the leaflet and smartphone app version (Supplementary Figure S1) were written in simple lay language with simple body illustrations of the placement of symptoms. The app contained the same information as the leaflet but was more detailed and with an audio function to accommodate women with low levels of literacy. Both the leaflet and app were available in six languages: in addition to Danish, in English, Arabic, Somali, Turkish, and Urdu as they represented the main languages of the largest ethnic minority groups in Denmark by the time of the trial [36]. The leaflet was distributed to all pregnant women at the first midwife ANC visits. A laminated version of the leaflet was available at ANC facilities to guide the dialogue about symptoms of pregnancy complications.

The leaflet included two unique codes for downloading the app: one for the woman and one for her partner. The codes could only be used once. All codes were individually linked to the participating maternity wards and enabled a direct dial function to the local maternity ward. Distribution of the leaflet and app was initiated once all midwives had attended the training session.

## 3. Materials and Methods

Supplementary Table S1 illustrates the elements of the process evaluation, the research questions, and data sources.

### 3.1. Implementation

Implementation was analyzed by assessing reach, dose, adaptations, and fidelity.

#### 3.1.1. Reach and Dose

Reach is the extent to which the target audience comes into contact with the intervention [26]. It was defined as the proportion of midwives from the ten intervention wards participating in the training sessions and the proportion of pregnant women receiving the leaflet. Participant lists documented the number of midwives attending the training session from each cluster. This information was compared to data on the number of midwives working in an ANC facility at each cluster. The number of women receiving the leaflet was assessed using cross-sectional survey data that included questions regarding the use of the MAMAACT leaflet and app (only given to women from the intervention sites) and collected through telephone interviews in six languages (Danish, English, Arabic, Turkish, Somali, and Urdu) by trained, bilingual interviewers after the implementation of the intervention from May to July 2019. The target was to recruit 150 women, including 30 women of non-Western origin for each cluster.

Dose refers to how much of the intervention is implemented [26]. Dose delivered was assessed by administrative data on the number of MAMAACT app downloads in relation to the number of received printed leaflets used as an approximation to the number of leaflets handed out at intervention sites. Information on reasons for non-use of the app and whether or not the leaflet and app provided new information about body signals to use during pregnancy was collected as part of the post-implementation survey.

#### 3.1.2. Fidelity and Adaptations

Fidelity is defined as the consistency of whether the intervention was implemented as planned. Adaptations are any alterations made to the intervention in the implementation process [26]. Fidelity was assessed using data from the dialogue meetings to assess how midwives integrated knowledge from the training session and the health education material in their daily practice. The local project midwives used a standardized information sheet to report back to the project team after each meeting. Using the logic model as an analytical framework, the information sheets were analyzed as qualitative data inspired by systematic Text Condensation [37]; reading all material to get an overview, identifying and coding of meaning bearing units, condensing and summarizing the findings.

### 3.2. Mechanisms of Impact

We previously analyzed pregnant women’s and midwives’ responses to the intervention using qualitative data (participant observations and field notes from 40 ANC visits, nine focus group interviews with midwives, and 21 in-depth individual interviews with non-Western immigrant women) collected from five of the ten intervention maternity wards in an article by Johnsen et al. [31]. In the present study, we have extracted perspectives from this article to study mechanisms of impact, i.e., how midwives and women experienced their participation in the intervention and how this affected their communication concerning response to pregnancy complications.

### 3.3. Context

The context was defined as everything external to the intervention that may act as a barrier or facilitator to its implementation [26]. Using the logic model, we delimited this process to focus on the most important organizational factors of ANC and women’s everyday life context that hindered or facilitated the use of the intervention. In two other previous articles by Johnsen et al. [30,32] these contextual aspects were analyzed using the same data from five intervention sites as in Johnsen et al. [31]. In the present study, we have extracted perspectives from these studies [30,32] to identify the most important contextual factors affecting the intervention implementation.

Further, in an information sheet, project and managing midwives from all 19 maternity wards reported the organization of ANC to inform about any targeted care for pregnant immigrant women, the interpretation services, and other ongoing research projects affecting the communication between midwives and pregnant women. This information was summed up and assessed for organizational differences and concurrent research having the potential to affect the outcomes of the intervention.

## 4. Results

### 4.1. Implementation

#### 4.1.1. Reach and Dose

In total, 346 midwives (87% of midwives working in ANC at the intervention sites) attended the training session in intercultural communication and cultural competence.

Table 1 shows the percentages of women in the MAMAACT post-implementation survey having received the MAMAACT leaflet. Of the 1304 women from intervention sites included in the survey, approximately 80% (n = 1051) received the MAMAACT leaflet. The amount was similar among women with non-Western immigrant backgrounds (80.2%) and a little higher among women with low educational levels (86%). Out of the 1051 women receiving the leaflet, 23% (n = 251) downloaded the app. Among non-Western immigrant women, the number was similar (22%) and among women with low education levels, only 18% downloaded the app. The main reasons for not downloading the app were forgetfulness and irrelevance: Lost or did not read the leaflet (24%), Felt no need for the app (21%), Did not know there was an app (18%), and Forgot to download the app (17%). Among women receiving the leaflet, 48% answered ‘yes’, while 25% answered ‘no’ to receiving information about body signals that they had been able to use during pregnancy and 24% had not read the leaflet. In the group of non-Western immigrant women, 62% answered ‘yes’ and 52% among women with low education levels to receiving information about body signals that they had been able to use during pregnancy. The tendency was similar among women who had downloaded the app. Of the 251 women downloading the app, 55% claimed to have been provided with usable information about body signals. The number was 73% among non-Western immigrant women and 83% among women with low education levels. Data stratified by intervention site are presented in Supplementary Table S2.

Table 2 shows that 13% of women and 4% of their partners downloaded the app in the period November 2018 to May 2020 in relation to the total number of received printed leaflets. The app was most often downloaded in Danish, English, and Arabic among both women and their partners; the percentage of downloads in foreign languages varied by intervention site (Supplementary Table S3).

By randomization, many of the largest maternity wards were intervention wards. Thus, knowing that 80% of the women from the survey received the MAMAACT leaflet, we expect that more than 20,000 women have received the intervention.

#### 4.1.2. Fidelity and Adaption

**Figure 2:**
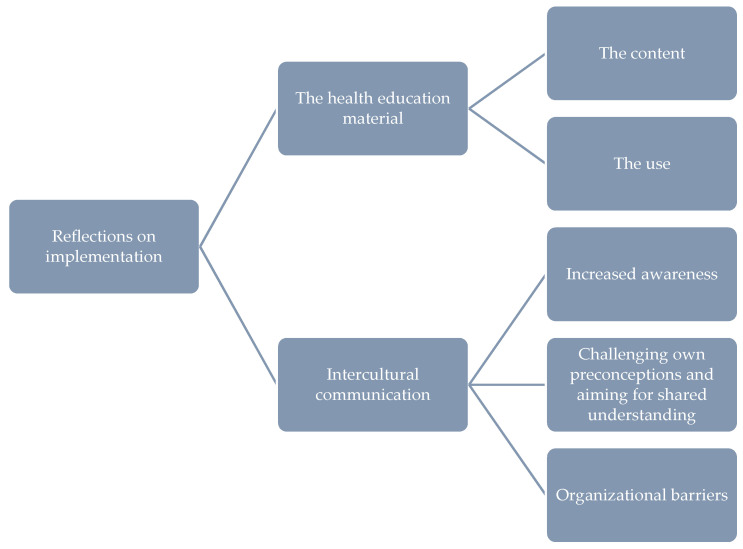
Analysis of the implementation reflections from the dialogue meetings with midwives (main and subcategories).

The analysis of the dialogue meetings focused on the health education material and the training session and ended with two categories and five subcategories (Figure 2). In the first round, 34 dialogue meetings were conducted, including 272 midwives, and in the second round, 30 meetings were conducted including 174 midwives.

Overall, the project midwives reported that the MAMAACT leaflet was meaningful as it was easy to use, concise, had the right level of detail, and focused on the right symptoms. However, in few meetings, the illustrations were expressed to be unclear and two symptoms (The baby’s movements and When the skin itches) too unspecific. The midwives expressed that they felt more confident that the women had understood the information about how to respond to symptoms during pregnancy when using the material and that it opened up for good conversations about body symptoms. The midwives reported that they distributed the leaflet to all women at their first midwifery ANC visit. It was emphasized that it was very useful in communications with immigrant and socially vulnerable women and less important for women with many resources. From a meeting it was written:

*“… it is a great supplement to the most insecure women. For the more resourceful it seems too simple”*

(W1, meeting 1.3).

The midwives used the material to a lesser extent at subsequent visits, where it primarily was included if women experienced symptoms. One reason was that the midwives did not wish to focus on risks, but on pregnancy as a natural process. The language options of the material were highly appreciated and the midwives felt it was an acknowledgment of the women’s needs. That the material was only available in six languages was demotivating for the midwives at specific hospitals with many women speaking different languages. Overall, the midwives were uncertain about how women used the materials after it was distributed. Nevertheless, from a few clusters, it was reported that pregnant women had used the material to request acute care for a relevant symptom (four cases).

It was clear that many of the midwives had become more aware of their communication with women from ethnic minority backgrounds. This focus from the training made the midwives express challenges faced in achieving needs-based communication. Some expressed that it could be difficult to build rapport and make women open up:

*“They (the midwives) experience a lot of one-way communication”*

(W6, meeting 1.2).

Sometimes the ethnic minority pregnant women were explained as being very timid and shy, while other times differences in knowledge about reproductive systems, expression of pain, and expectations to ANC were experienced as difficult to overcome. Some midwives found it challenging to assess the women’s understanding without asking too simple questions that made the midwives feel unintendedly condescending. All these challenges were enlarged in the case of language barriers. The midwives had many examples of misunderstandings. Many midwives expressed that the MAMAACT intervention had made them more reflective about their cultural understandings and preconceptions about the women. They found themselves to be more curious to get to know the women, their situation, past experiences, health literacy levels, and perceptions about body and health. They tried not to anticipate and categorize the women, but to be open-minded, patient, and focused on the individual:

*“The midwives express more awareness about preconceptions and less stigmatization”*

(W4, meeting 1.1).

Some midwives expressed to be more careful to express themselves clearly and use more open-ended questions, active listening and had the woman explain back her understanding. Concurrently, resistance was also expressed at a few dialogue meetings, as the midwives mentioned that nothing had changed in their ways of communication, as there was no need to. 

It was a general concern that overcoming cultural and language barriers was a difficult task hindered by organizational barriers. Time pressure, lack of flexibility in planning the visits, poor quality of interpretation services, and standardized criteria for the content of the visits made it very difficult for the midwives to act on their reflections, some even found it utopic to build a relation that would make the most vulnerable women open up:

*“The important things we would like to come around drown in those things we have to do KRAM (assessment of diet, smoking, alcohol and physical activity), urine tests, etc.”*

(W7, meeting 2.5).

The reflections about intercultural communication were much more pronounced and elaborated in the reports from the second round of dialogue meetings, which could be due to either a development over time or a result of the changed manual for the second round meetings.

### 4.2. Mechanisms

From the article by Johnsen et al. about midwives’ and non-Western pregnant women’s attitudes of and experiences with the intervention [31], it was likewise found that the MAMAACT health education material was easy to implement and that the training session and dialogue meetings had initiated more reflections about intercultural communication among midwives and improved the awareness about symptoms of pregnancy complications and health system navigation among women.

At home, women used the leaflet and app to distinguish between normal and abnormal symptoms. Having the leaflet and app in their native language was described to increase their access to information and some women would use the app to learn Danish words related to pregnancy and birth. Observations showed that most midwives would only use a few minutes to introduce the material and follow-up on the material was low. According to the midwives, this was due to competing tasks. Midwives used the most time to inform women about their pregnancy, and women primarily talked about their symptoms when invited to do so. Thus, the analysis indicated that the mechanisms of increasing needs-based communication and active engagement during ANC visits seemed challenged by organizational barriers and habitual interaction patterns.

### 4.3. Contextual Factors

The article by Johnsen et al. analyzing implementation barriers [32] found that information exchange between general practitioners and midwives was extremely limited and delayed differentiated care or interpreter services at the midwifery ANC. Varying quality of interpreter services and frequently no options for extending ANC time when using interpreter assistance challenged mutual understanding between midwives and non-Danish speaking women. In addition, the analysis showed that restricted time schedules and high task loads made it difficult for the midwives to implement the project as intended regarding achieving needs-based communication [32].

Individual interviews about non-Western women’s everyday life situations analyzed by Johnsen et al. showed that women’s sources of information often were the internet due to lack of network, considering pregnancy a private matter, or protection of family and relatives for involvement in worrisome issues [30]. Further, some women expressed previous negative experiences with healthcare encounters and therefore kept interactions with maternity care providers at a minimum. Women described having many domestic responsibilities resulting in limited action space for self-care. Also, some women were unaware of their rights and feared the possible economic consequences of seeking medical assistance during work or school hours. These circumstances were likely to reduce the women’s use of the MAMAACT health education material and delay women in seeking healthcare for acute symptoms [30].

From the organizational information sheets, it was apparent that the national policy for ANC was implemented differently at the 19 maternity wards. At ten wards, they provided immigrant targeted care, where immigrant women would be seen by a specific midwife having extended consultation time. These ten clusters were evenly distributed between the intervention and control group, and thus should not bias the analysis of effects. All clusters ensured interpreter services to women with low Danish language proficiency. However, only six of the 19 clusters reported having the option of extending the consultation time, when interpretation was needed. Five of these clusters allowing more time were in the control group, and thus the implementation of the intervention potentially occurred in a context of intensified time pressure for women with low Danish language proficiency. Among the reports of other ongoing research projects, only one was considered to potentially affect the outcomes of the MAMAACT intervention. This was a non-intervention project aiming to better understand the development of preeclampsia (PRESIDE). In PRESIDE, women had an additional ultrasound scan of the blood flow in the uterine artery and received informed consent material (in Danish only) about preeclampsia [38]. As warning signs of preeclampsia were included in the MAMAACT material and the inclusion of women to PRESIDE started at one ward in the last months of the MAMAACT implementation, some overlap in effects on women’s knowledge about preeclampsia might have happened.

## 5. Discussion

### 5.1. Main Findings and Interpretations

This process evaluation aimed to explore the implementation process of the MAMAACT intervention to examine which elements were implemented and how, to examine mechanisms of change among pregnant women and midwives as well as the influence of contextual factors. The results showed that more than 80% of women had received the leaflet during ANC visits, indicating a reach of more than 20,000 women. Around half of the women receiving the leaflet found the content useful and midwives evaluated the material as especially useful in communications with non-Danish speaking and socially vulnerable women and less relevant for resourceful women. Overall, the app was used more selectively by both pregnant women and their partners. However, among the specific target groups of non-Western immigrant women and women with low educational levels, a larger proportion evaluated it as useful. The audio function included in the app sought to accommodate the needs of women with low levels of literacy. Thus, it is encouraging that the material was evaluated most useful among the group of women intended to benefit the most.

The reach of the post-graduate training of midwives in intercultural communication and competence (training session and dialogue meetings) was as high as 87%. The training increased the awareness and reflections on their communication with women from various social and cultural backgrounds. Organizational barriers including lack of time, flexibility in ANC visits, and poor quality of interpreter services as well as habitual interaction patterns made it challenging for midwives to change practice and make room for more needs-based communication. Thus, the evaluation of intervention effects will show whether insights from the training sessions and dialogue meetings despite contextual barriers have supported more needs-based interactions during ANC visits. If so, this might increase women’s health literacy levels (primary trial outcome) as an indication of increased health literacy responsiveness by the midwives.

Women of non-Western immigrant backgrounds used the materials at home to distinguish between normal and abnormal symptoms of pregnancy complications [31]. However, obstacles in everyday life including lack of social network and domestic responsibilities as well as previous negative experiences with the maternity care system seemed to lower the level of healthcare-seeking in case of symptom doubt [30].

Based on the analysis, no organizational changes or other ongoing research projects at intervention and control sites seemed to potentially affect the evaluation of the primary trial outcome, except PRESIDE at one ward, which should be taken into account in the effectiveness analyses.

Other current intervention studies are aiming to improve communication and interaction in maternity care to prevent adverse pregnancy outcomes in vulnerable groups including immigrants. These include group-based ANC [39,40] and doula support to immigrant women [41]. However, to our knowledge, no studies have focused on improving the management of pregnancy complications and literature on ANC interventions specifically targeting socially disadvantaged women is sparse.

A recent study, being part of a larger project ‘Operational Refugee and Migrant Maternal Approach’ (ORAMMA), examined the impact of culturally sensitive maternity care training for midwives in areas of the UK, the Netherlands, and Greece. The training proved to impact midwives’ knowledge and self-perceived cultural competence [42]. However, in line with our results, the midwives reported several contextual challenges in caring for immigrants. These included problems with no-shows at ANC visits due to issues with distance to travel and childcare as well as a need for more time during visits in the presence of language barriers [42].

Using the logic model to elaborate the hypothesis of mechanisms and the role of context made us understand the most important mechanisms and barriers to potential effects. Our analysis shows that the role of context did not vary much across the maternity wards in Denmark despite the differences in the organization of ANC, work culture among midwives, and characteristics of the women in the different settings across Denmark. This is rather surprising, however, it might relate to the flexibility in the implementation and the overall fit with the national Danish policy for ANC [34]. Further, both the intervention and the analysis were guided by theories of health literacy, cultural competence, and cultural health capital and thus aimed for a theoretical understanding of the driving mechanisms of change. It is still too early to conclude about effects in women’s health literacy levels and management of pregnancy complications; however, results of this study suggest that the current version of the MAMAACT intervention had difficulties in profoundly changing the interaction patterns towards more needs-based ANC. Likewise, a review of cultural competence interventions recently concluded that the effects of cultural competence training of healthcare providers can improve providers’ knowledge and attitudes, however, it was not possible to document effects on patient health outcomes [43], suggesting barriers in translating knowledge into practice. In a Cochrane review, the knowledge base on cultural competence interventions was found vague and inconsistent, due to inconsistency in the use of the term cultural competence and low quality of the evaluations [44]. Both reviews point to cultural competence interventions as a means to overcome inequity in healthcare, but state that system-wide approaches are needed. As emphasized by Seeleman et al., healthcare providers can only act within the limits of the organization [16]. Thus, for interventions to effectively promote responsiveness to diversity they should focus not only on the cultural competence of the individual level but just as important at the organizational and systemic level [45,46]. Further, from an organizational theoretical perspective, it is acknowledged that organizational readiness including the level of organizational support, motivation, and commitment is essential for program implementation and change of practices [47]. The high proportion of managing midwives joining the MAMAACT study (19 managers of 20) indicate large commitment and motivation from the midwifery management level; however, the scope of the intervention did not enable changes at these structural levels. Further efforts at the organizational level to enable more time and flexibility during ANC visits for midwives to adapt to new ways of communicating and caring for the increasingly diverse group of pregnant women seem warranted. Including elements of intercultural communication and cultural competence in the education program of new midwives might be a way forward to introduce reflections and awareness at an early stage.

### 5.2. Strengths and Limitations

The large proportion of maternity wards in Denmark joining the cluster randomized trial and the high reach of the intervention are major strengths and indicate the clinical relevance of the intervention. Another main strength is the combination of both quantitative and qualitative data from various sources to evaluate the implementation process of the intervention [26]. In general, there was consistency in the themes emerging from the dialogue meetings and the previous qualitative studies by Johnsen et al. This occurred even though the data from the dialogue meetings included all ten intervention facilities and were gathered by local project midwives during the entire implementation process in contrary to the in-depth data from five selected sites collected by one of the MAMAACT researchers. In addition, the qualitative data support survey data concerning the usage of the leaflet and app, providing reliability to the study results. We find it a strength that we have conducted the process evaluation before the analysis of intervention outcome effects to stay unbiased, to guide potential sub-analyses, and to allow insights from the process evaluation to assist in interpreting the observed intervention effects [26]. A limitation is the lack of data on the number of leaflets handed out at each intervention site. Due to this, the percentage of app downloads in relation to the number of received printed leaflets in each language is a rough estimate. However, the exact number of handouts will be lower than the total number of leaflets received, why the percentages of app downloads might be underestimated. Survey data, however, confirm the overall limited use of the app. The representativeness of non-Western immigrant women in survey data was high and the largest country groups of non-Western immigrants in the survey data matched the distribution among the total of non-Western immigrant women giving birth in Denmark in 2019 [36,48]. The representativeness of women with social disadvantages was low in the quantitative data which could be a consequence of the sampling strategy had focused on the recruitment of non-Western immigrant women. Thus, there is less power to evaluate the implementation process and the forthcoming effects within this subgroup. Inclusion criteria for the women participating in the in-depth interviews and observations were being of non-Western origin [30,31,32]; however, the group was also characterized by having low socioeconomic status. In the qualitative analysis, the interactions between ethnicity and socioeconomic position were fundamental for the analysis of the experience with MAMAACT.

## 6. Conclusions

This process evaluation showed that the reach of the MAMAACT intervention was high and that it was well-received among midwives and pregnant women of whom approximately half found the content of the leaflet useful. The app was used more selectively but evaluated most useful among the specific target groups of non-Western immigrants and socially disadvantaged women. The midwives distributed the leaflet to all women at the first ANC visit as intended, but follow-up was low. The training session and dialogue meetings with a focus on intercultural communication and competence increased midwives’ awareness and reflections in care provision for immigrant women. However, organizational factors caused difficulties in changing the interaction patterns during ANC visits towards more needs-based communication. Thus, when interpreting the intervention effects, attention should be drawn to both organizational and interpersonal factors in the clinic as well as the pregnant women’s life situations. These insights into the implementation process of the MAMAACT intervention contribute to the understanding of what factors might have enabled or hindered the intended mechanisms and is important knowledge for the future interpretation of intervention effects.

## Figures and Tables

**Table 1:** Process evaluation questions on the use of the MAMAACT leaflet and app from the MAMAACT post-implementation cross-sectional survey, 2019.

	Women with Low-Level Education ^a^ N = 78 (100)	Non-Western Immigrant N = 217 (100)	Total N = 1304 (100)
**Did the midwife give you the leaflet “MAMAACT”?**			
*Yes*	67 (85.9)	174 (80.2)	1051 (80.6)
*No*	11 (14.1)	38 (17.5)	227 (17.4)
*Unanswered*	0 (0.0)	5 (2.3)	26 (2.0)
**Did the leaflet provide you with information about body signals that you have been able to use during your pregnancy? ^b^**			
*Yes*	35 (52.2)	111 (62.0)	522 (48.5)
*No*	16 (23.9)	31 (17.3)	266 (24.7)
*Have not read the leaflet*	14 (20.9)	32 (17.9)	264 (24.5)
*Unanswered*	2 (3.0)	5 (2.8)	25 (2.3)
**Did you download the app “MAMAACT”? ^b^**			
*Yes*	12 (17.9)	40 (22.3)	251 (23.3)
*No*	55 (82.1)	135 (75.4)	811 (75.3)
*Unanswered*	0 (0.0)	4 (2.3)	15 (1.4)
**Why did you not download the app? You can choose between one of the following four options: ^c^**			
*The* *MAMAACT leaflet was sufficient*	10 (18.2)	34 (24.5)	174 (21.1)
*You did not have a smartphone to install it on*	1 (1.8)	2 (1.4)	12 (1.5)
*It was difficult to find or install*	5 (9.1)	12 (8.6)	15 (1.8)
*It was irrelevant; please elaborate*	35 (63.6)	80 (57.6)	595 (72.0)
*Unanswered*	4 (7.3)	11 (7.9)	30 (3.6)
**It was irrelevant; please elaborate ^d^**			
*Forgot to download the app*	8 (22.9)	14 (17.5)	102 (17.1)
*Did not use the app due to technical problems*	0 (0.0)	1 (1.3)	10 (1.7)
*Woman herself or partner works within healthcare*	0 (0.0)	1 (1.2)	12 (2.0)
*Lost or did not read the leaflet*	5 (14.3)	18 (22.5)	145 (24.4)
*Did not have time/surplus to get acquainted with the app*	0 (0.0)	1 (1.2)	12 (2.0)
*Use other apps*	1 (2.8)	6 (7.5)	35 (5.9)
*Felt no need for the app (partly due to knowledge from previous pregnancy)*	7 (20.0)	14 (17.5)	125 (21.0)
*Did not know there was an app*	8 (22.9)	19 (23.8)	111 (18.7)
*Other reasons*	6 (17.1)	6 (7.5)	43 (7.2)
**Did the app provide you with information about body signals that you have been able to use during your pregnancy? ^e^**			
*Yes*	10 (83.3)	32 (72.7)	147 (55.3)
*No*	1 (8.3)	2 (4.6)	56 (21.0)
*Have not used the app*	1 (8.3)	6 (13.6)	47 (17.7)
*Unanswered*	0 (0.0)	4 (9.1)	16 (6.0)

^a^ Low-level education is defined by women with primary education or lower as their highest attained educational level. ^b^ Percentages are based on the total of women answering ‘yes’ to or not answering the question: Did the midwife give you the leaflet “MAMAACT”? ^c^ Percentages are based on the total of women answering ‘no’ to or not answering the question: Did you download the app “MAMAACT”? ^d^ Percentages are based on the total of women answering ‘It was irrelevant; please elaborate’ to the question: Why did you not download the app? ^e^ Percentages are based on the total of women answering ‘yes’ to or not answering the question: Did you download the app “MAMAACT”?

**Table 2:** The total number of received printed MAMAACT leaflets and app downloads by language from November 2018 to May 2020.

	Number of Printed Leaflets	App Downloads (Woman) ^a^	App Downloads (Partner) ^a^
Danish	29,640	4184 (14.1)	1145 (3.9)
English	3350	527 (15.7)	174 (5.2)
Arabic	1570	130 (8.3)	39 (2.5)
Turkish	810	34 (4.2)	9 (1.1)
Somali	790	13 (1.6)	3 (0.4)
Urdu	790	26 (3.3)	9 (1.1)
Total	36,950	4914 (13.3)	1379 (3.7)

^a^ Numbers in parenthesis are the percentages of app downloads in relation to the number of received printed leaflets in the same language.

## Data Availability

The qualitative data are confidential according to the General Data Protection Regulation and cannot be provided. Quantitative datasets can be available upon reasonable request on an aggregated level to protect individual data.

## Supplementary Materials

The following are available online at https://www.mdpi.com/article/10.3390/ijerph18168583/s1, Figure S1: The MAMAACT app in English, Table S1: Process evaluation components, research questions and data sources, Table S2: Process evaluation questions on the use of the MAMAACT leaflet and app per cluster in the intervention group, from the MAMAACT post-implementation cross-sectional survey, 2019, Table S3: The total number of received printed MAMAACT leaflets and app downloads by language and per cluster in the intervention group from November 2018 to May 2020.
