# Supplementary material for: Implementation, Mechanisms and Context of the MAMAACT Intervention to Reduce Ethnic and Social Disparity in Stillbirth and Infant Health
Source: Int J Environ Res Public Health. 2021 Aug 14;18(16):8583. doi: 10.3390/ijerph18168583 (PMC8391863; doi:10.3390/ijerph18168583)
Supplement: Supplementary file 1 [file ijerph-18-08583-s001.zip › ijerph-1312252-supplementary/Table S1.pdf]

Table S1. Process evaluation components, research questions and data sources

	Research questions	Data sources
**Implementation**		
Reach	What proportion of antenatal care (ANC) midwives attended the training session?	Lists of ANC midwives and participant lists
	How many midwives attended the dialogue meetings?	Project midwives records from all dialogue meetings
	How many women received the leaflet?	Survey data
Dose	How many leaflets were given to each hospital?	Administrative data on the number of received printed leaflets
	Did the leaflet provide new information about body signals?	Survey data
	How many downloaded the app?	Survey data and administrative data on app downloads
	What were the main reasons for not downloading the app?	Survey data
	Did the app provide new information about body signals?	Survey data
Fidelity	How did midwives reflect on the integration of knowledge gained from the training session into daily practice?	Project midwives records from all dialogue meetings
	What did midwives discuss?<br>How did they discuss the use of the leaflet in daily practice?	Project midwives records from all dialogue meetings
	How did midwives use the leaflet in daily practice to talk about body symptoms of pregnancy complications?	Sum-up of a qualitative study by Johnsen et al. 2021 [31]
**Mechanisms of impact**		
Participant responses to the intervention	What were the midwives' experiences with the training session?	Sum-up of a qualitative study by Johnsen et al. 2021 [31]
	What were the midwives' experiences with the dialogue meetings?	Sum-up of a qualitative study by Johnsen et al. 2021 [31]
	How did women react to the MAMAACT leaflet and app?	Sum-up of a qualitative study by Johnsen et al. 2021 [31]
**Contextual factors**		
	Where there other important processes affecting ANC at interventions and control sites affecting communication on pregnancy warning signs during the trial period?	Information sheets from maternity wards on organizational inputs and other research projects
	How do barriers in non-Western immigrant women's everyday life situations affect responses to pregnancy complications?	Sum-up of a qualitative implementation study by Johnsen et al. 2020 [30]
	How did main organizational barriers impact the intended mechanisms?	Sum-up of a qualitative implementation study by Johnsen et al. 2020 [32]
