# Supplementary material for: Implementation, Mechanisms and Context of the MAMAACT Intervention to Reduce Ethnic and Social Disparity in Stillbirth and Infant Health
Source: Int J Environ Res Public Health. 2021 Aug 14;18(16):8583. doi: 10.3390/ijerph18168583 (PMC8391863; doi:10.3390/ijerph18168583)
Supplement: Supplementary file 1 [file ijerph-18-08583-s001.zip › ijerph-1312252-supplementary/Table S2.pdf]

Supplementary table S2. Process evaluation questions on the use of the MAMAACT leaflet and app per cluster in the intervention group, from the MAMAACT post-implementation cross-sectional survey, 2019

	**W1[a]** N=135 (100)	**W2[a]** N=93 (100)	**W3[a]** N=117 (100)	**W4[a]** N=156 (100)	**W5[a]** N=120 (100)	**W6[a]** N= 125 (100)	**W7[a]** N= 124 (100)	**W8[a]** N=122 (100)	**W9[a]** N=156 (100)	**W10[a]** N=156 (100)
**Did the midwife give you the leaflet "MAMAACT"?**										
Yes	114 (84.4)	84 (90.3)	87 (74.4)	123 (78.8)	86 (71.7)	92 (73.6)	109 (87.9)	101 (82.8)	127 (81.4)	128 (82.1)
No	19 (14.1)	6 (6.5)	25 (21.4)	31 (19.9)	31 (25.8)	29 (23.2)	15 (12.1)	18 (14.7)	26 (16.7)	27 (17.3)
Unanswered	2 (1.5)	3 (3.2)	5 (4.3)	2 (1.3)	3 (2.5)	4 (3.2)	0 (0.0)	3 (2.5)	3 (1.9)	1 (0.6)
**Did the leaflet provide you with information about body signals that you have been able to use during your pregnancy? [b]**										
Yes	64 (55.2)	38 (43.7)	45 (48.9)	66 (52.8)	36 (40.5)	40 (41.7)	58 (53.2)	46 (44.2)	60 (46.2)	69 (53.5)
No	31 (26.7)	30 (34.5)	18 (19.6)	19 (15.2)	25 (28.1)	28 (29.2)	23 (21.1)	24 (23.1)	34 (26.2)	34 (26.4)
Have not read the leaflet	19 (16.4)	13 (14.9)	25 (27.2)	39 (31.2)	26 (29.2)	26 (27.1)	27 (24.8)	30 (28.9)	34 (26.1)	25 (19.4)
Unanswered	2 (1.7)	6 (6.9)	4 (4.4)	1 (0.8)	2 (2.2)	2 (2.1)	1 (0.9)	4 (3.8)	2 (1.5)	1 (0.7)
Missing	19	6	25	31	31	29	15	18	26	27
**Did you download the app "MAMAACT"? [b]**										
Yes	33 (28.4)	44 (50.6)	23 (25.0)	24 (19.2)	17 (19.1)	10 (10.4)	19 (17.4)	13 (12.5)	33 (25.4)	35 (27.1)
No	82 (70.7)	41 (47.1)	65 (70.6)	101 (80.8)	70 (78.6)	85 (88.5)	90 (82.6)	88 (84.6)	95 (73.1)	94 (72.9)
Unanswered	1 (0.9)	2 (2.3)	4 (4.4)	0 (0.0)	2 (2.3)	1 (1.1)	0 (0.0)	3 (2.9)	2 (1.5)	0 (0.0)
Missing	19	6	25	31	31	29	15	18	26	27
**Why did you not download the app? You can choose between one of the following four options: [c]**										
The MAMAACT leaflet was sufficient	21 (25.3)	6 (14.0)	7 (10.1)	22 (21.8)	15 (20.8)	16 (18.6)	25 (27.8)	17 (18.7)	22 (22.6)	23 (24.5)
You did not have a smartphone to install it on	1 (1.2)	1 (2.3)	3 (4.3)	1 (1.0)	0 (0.0)	1 (1.2)	1 (1.1)	1 (1.1)	1 (1.0)	2 (2.1)
It was difficult to find or install	1 (1.2)	1 (2.3)	3 (4.4)	2 (2.0)	2 (2.8)	0 (0.0)	2 (2.2)	1 (1.1)	1 (1.0)	2 (2.1)
It was irrelevant; please elaborate	55 (66.3)	31 (72.1)	51 (73.9)	76 (75.2)	51 (70.8)	66 (76.7)	61 (67.8)	69 (75.8)	69 (71.1)	66 (70.2)
Unanswered	5 (6.0)	4 (9.3)	5 (7.3)	0 (0.0)	4 (5.6)	3 (3.5)	1 (1.1)	3 (3.3)	4 (4.1)	1 (1.1)
Missing	52	50	48	55	48	39	34	31	59	62
**It was irrelevant; please elaborate [d]**										
Forgot to download the app	8 (14.6)	8 (25.8)	9 (17.6)	16 (21.0)	6 (11.8)	13 (19.7)	7 (11.5)	13 (18.8)	13 (18.8)	9 (13.6)
Did not use the app due to technical problems	1 (1.8)	2 (6.5)	0 (0.0)	1 (1.3)	0 (0.0)	1 (1.5)	2 (3.3)	1 (1.5)	0 (0.0)	2 (3.0)
Woman herself or partner works within healthcare	1 (1.8)	0 (0.0)	1 (2.0)	0 (0.0)	1 (2.0)	3 (4.6)	1 (1.6)	1 (1.5)	1 (1.5)	3 (4.6)
Lost or did not read the leaflet	9 (16.4)	4 (12.9)	14 (27.5)	19 (25.0)	15 (29.4)	18 (26.9)	15 (24.6)	15 (21.7)	19 (27.5)	17 (25.8)
Did not have time/ surplus to get acquainted with the app	0 (0.0)	2 (6.4)	1 (2.0)	1 (1.3)	3 (5.9)	2 (3.0)	0 (0.0)	0 (0.0)	3 (4.4)	0 (0.0)
Use other apps	3 (5.5)	2 (6.5)	4 (7.8)	5 (6.6)	2 (3.9)	5 (7.6)	2 (3.3)	5 (7.3)	4 (5.8)	3 (4.6)
Felt no need for the app (partly due to knowledge from previous pregnancy)	13 (23.6)	8 (25.8)	10 (19.6)	14 (18.4)	12 (23.5)	12 (18.2)	14 (22.9)	9 (13.0)	14 (20.3)	19 (28.8)
Did not know there was an app	16 (29.1)	3 (9.7)	8 (15.7)	17 (22.4)	9 (17.6)	7 (10.6)	16 (26.2)	18 (26.1)	10 (14.5)	7 (10.6)
Other reasons	4 (7.2)	2 (6.4)	4 (7.8)	3 (4.0)	3 (5.9)	5 (7.6)	4 (6.6)	7 (10.1)	5 (7.2)	6 (9.1)
Missing	80	62	66	80	69	59	63	53	87	90
**Did the app provide you with information about body signals that you have been able to use during your pregnancy? [e]**										
Yes	23 (67.7)	14 (30.4)	17 (63.0)	14 (58.3)	8 (42.1)	6 (54.5)	11 (57.9)	11 (68.8)	20 (57.1)	23 (65.7)

No	7 (20.6)	17 (37.0)	3 (1.1)	5 (20.8)	4 (21.1)	2 (18.2)	5 (26.3)	0 (0.0)	8 (22.9)	5 (14.3)
Have not used the app	3 (8.8)	13 (28.3)	3 (1.1)	5 (20.8)	3 (15.8)	2 (18.2)	3 (15.8)	3 (18.7)	5 (14.3)	7 (20.0)
Unanswered	1 (2.9)	2 (4.3)	4 (14.8)	0 (0.0)	4 (21.0)	1 (9.1)	0 (0.0)	2 (12.5)	2 (5.7)	(0.0)

[a] Anonymized names of the ten maternity wards (clusters) included in the intervention group
[b] Percentages are based on the total of women answering 'yes' to or not answering the question: Did the midwife give you the leaflet "MAMAACT"?
[c] Percentages are based on the total of women answering 'no' to or not answering the question: Did you download the app "MAMAACT"?
[d] Percentages are based on the total of women answering 'It was irrelevant; please elaborate' to the question: Why did you not download the app?
[e] Percentages are based on the total of women answering 'yes' to or not answering the question: Did you download the app "MAMAACT"?
