# Supplementary material for: Implementation, Mechanisms and Context of the MAMAACT Intervention to Reduce Ethnic and Social Disparity in Stillbirth and Infant Health
Source: Int J Environ Res Public Health. 2021 Aug 14;18(16):8583. doi: 10.3390/ijerph18168583 (PMC8391863; doi:10.3390/ijerph18168583)
Supplement: Supplementary file 1 [file ijerph-18-08583-s001.zip › ijerph-1312252-supplementary/Table S3.pdf]

Supplementary table S3. The total number of received printed MAMAACT leaflets and app downloads by language and per cluster in the intervention group from Nov. 2018 to May 2020

	W1[a]	W2[a]	W3[a]	W4[a]	W5[a]	W6[a]	W7[a]	W8[a]	W9[a]	W10[a]
**Number of received printed leaflets**										
Danish	2900	4500	1640	5600	3300	4000	1900	2300	1600	1900
English	300	400	250	700	300	450	200	350	200	200
Arabic	70	200	150	200	200	250	100	200	80	120
Turkish	50	50	100	180	50	100	70	80	80	50
Somali	50	80	100	130	50	100	70	80	80	50
Urdu	50	50	50	180	80	100	70	80	80	50
Total	3420	5280	2290	6990	3980	5000	2410	3090	2120	2370
**App downloads (woman)[b]**										
Danish	467 (16.1)	565 (12.6)	211 (12.9)	993 (17.7)	506 (15.3)	338 (8.5)	222 (11.7)	243 (10.6)	323 (20.2)	316 (16.6)
English	59 (19.7)	68 (17.0)	13 (5.2)	202 (28.9)	60 (20.0)	42 (9.3)	19 (9.5)	22 (6.3)	12 (6.0)	30 (15.0)
Arabic	13 (18.6)	15 (7.5)	10 (6.7)	20 (10.0)	27 (13.5)	12 (4.8)	5 (5.0)	12 (6.0)	6 (7.5)	10 (8.3)
Turkish	3 (6.0)	3 (3.8)	0 (0.0)	25 (13.9)	1 (2.0)	1 (1.0)	0 (0.0)	1 (1.3)	0 (0.0)	0 (0.0)
Somali	3 (6.0)	3 (3.8)	0 (0.0)	4 (3.1)	0 (0.0)	1 (1.0)	0 (0.0)	1 (1.3)	0 (0.0)	1 (2.0)
Urdu	1 (2.0)	0 (0.0)	0 (0.0)	21 (11.7)	2 (2.5)	1 (1.0)	1 (1.4)	0 (0.0)	0 (0.0)	0 (0.0)
Total	546 (16.0)	654 (12.4)	234 (10.2)	1265 (18.1)	596 (15.0)	395 (7.9)	247 (10.2)	279 (9.0)	341 (16.1)	357 (15.1)
**App downloads (partner)[b]**										
Danish	137 (4.7)	167 (3.7)	65 (4.0)	287 (5.1)	118 (3.6)	89 (2.2)	60 (3.2)	59 (2.6)	81 (5.1)	82 (4.3)
English	12 (4.0)	22 (5.5)	5 (2.0)	69 (9.9)	20 (6.7)	12 (2.7)	7 (3.5)	10 (2.9)	5 (2.5)	12 (6.0)
Arabic	4 (5.7)	5 (2.5)	4 (2.7)	7 (3.5)	6 (3.0)	5 (2.0)	1 (1.0)	5 (2.5)	1 (1.3)	1 (0.8)
Turkish	0 (0.0)	0 (0.0)	0 (0.0)	7 (3.9)	1 (2.0)	0 (0.0)	0 (0.0)	1 (1.3)	0 (0.0)	0 (0.0)
Somali	1 (2.0)	1 (1.3)	0 (0.0)	0 (0.0)	0 (0.0)	0 (0.0)	0 (0.0)	1 (1.3)	0 (0.0)	0 (0.0)
Urdu	1 (2.0)	0 (0.0)	0 (0.0)	4 (2.2)	3 (3.8)	1 (1.0)	0 (0.0)	0 (0.0)	0 (0.0)	0 (0.0)
Total	155 (4.5)	195 (3.7)	74 (3.2)	374 (5.4)	148 (3.7)	107 (2.1)	68 (2.8)	76 (2.5)	87 (4.1)	95 (4.0)

[a] Anonymized names of the ten maternity wards (clusters) included in the intervention group

[b] Numbers in parenthesis are the percentages of app downloads in relation to the number of received printed leaflets in the same language
