# Supplementary figures and images for: Implementation, Mechanisms and Context of the MAMAACT Intervention to Reduce Ethnic and Social Disparity in Stillbirth and Infant Health
Source: Int J Environ Res Public Health. 2021 Aug 14;18(16):8583. doi: 10.3390/ijerph18168583 (PMC8391863; doi:10.3390/ijerph18168583)

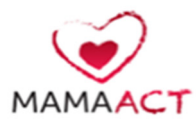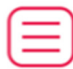

Select the symptom for more information

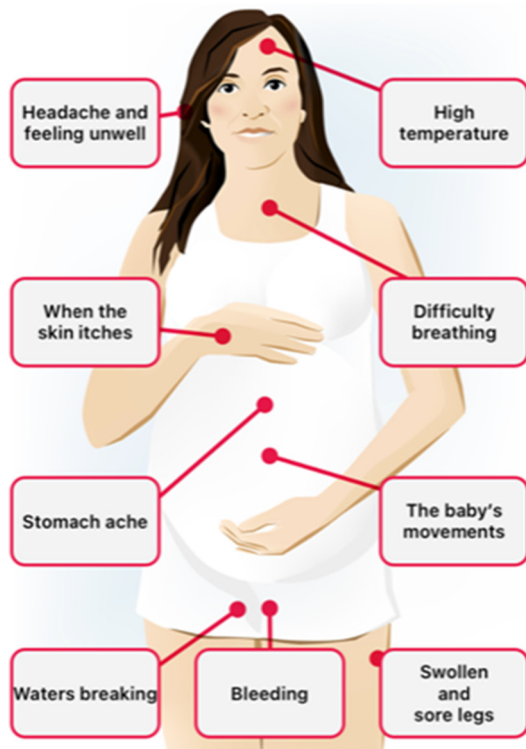

Supplementary Figure S1. The MAMA ACT app in English

Supplement: Supplementary file 1 [file ijerph-18-08583-s001.zip › ijerph-1312252-supplementary/Supplementary Figure S1.pdf]
